# Evaluating the Training the Trainer Course for Psychiatry Higher Trainees in the West Midlands

**DOI:** 10.1192/bjo.2024.318

**Published:** 2024-08-01

**Authors:** Meena Murugan, Bijal Sangoi, Feroz Nainar

**Affiliations:** 1 Birmingham Solihull Mental Health Foundation Trust, Birmingham, United Kingdom; 2 Midlands Partnership Foundation Trust, Stafford, United Kingdom; 3 Birmingham Community Healthcare Trust, Birmingham, United Kingdom

## Abstract

**Aims:**

The Royal College of Psychiatrists sets out ‘Education and Training’ as one of the High Level Outcomes (HLOs) in its GMC approved curriculum for higher speciality trainees in Psychiatry. The West Midlands (WM) School of Psychiatry runs a well-established 3-day Training the Trainer (TTT) course to support acquisition of teaching skills and key capabilities to help prepare trainees to become trainers as Consultants.

We aim to explore the views and attitudes held by WM Psychiatry higher trainees towards the current TTT course and other teaching opportunities available across the region.

**Methods:**

An anonymous online scoping survey was sent to all WM Psychiatry higher trainees, via Microsoft Forms, in January 2023. This comprehensive survey included questions on the trainees’ awareness of the TTT course and available teaching opportunities, as well as support and barriers in accessing these. We designed dichotomous, rating and free text questions to generate both quantitative and qualitative data.

**Results:**

Key findings of the survey included:

- 27 out of 40 trainees responded. All subspecialty training programmes were represented.
- Many trainees were aware of the WM TTT course (81%). No trainees had accessed private TTT courses.
- Most trainees felt the current available opportunities allowed them to meet the curriculum requirements (82%) and felt their supervisor could provide support in gaining teaching experiences (93%).
- Only two-thirds of trainees felt the current opportunities prepared them to be an effective Consultant trainer (67%). Some were also uncertain of teaching opportunities available in the deanery (41%).
- Trainees expressed a preference of learning through small group tutorials, interactive workshops and experiential learning.
- Trainees requested incorporating content around innovative technology in medical education including artificial intelligence and simulation as well as formal qualifications in medical education.

**Conclusion:**

The project has shown that the current TTT course is effective in supporting Psychiatry higher trainees meet their curriculum requirements, however there is a scope to adjust the content to meet their changing needs and align with digital advancements in medical education. We suggest the course should be delivered in a more interactive and engaging manner for example using breakout rooms and workshops. To ensure all trainees are aware of the course and teaching opportunities available, an information leaflet outlining the TTT course will be sent out as part of the induction process. It is hoped that with these improvements, the needs of Psychiatry higher trainees will be better met as they move forwards in their careers and become Consultant trainers.

